# Epidemiology of heart failure in Germany: a retrospective database study

**DOI:** 10.1007/s00392-017-1137-7

**Published:** 2017-07-26

**Authors:** Stefan Störk, Renate Handrock, Josephine Jacob, Jochen Walker, Frederico Calado, Raquel Lahoz, Stephan Hupfer, Sven Klebs

**Affiliations:** 1 Comprehensive Heart Failure Centre Würzburg and Department of Internal Medicine I, University and University Hospital Würzburg, Würzburg, Germany; 2 Novartis Pharma GmbH, Nuremberg, Germany; 3 Elsevier Health Analytics, Berlin, Germany; 4 Health Risk Institute, Berlin, Germany; 5 Novartis Pharma AG, Basel, Switzerland; 6 Deutsches Zentrum für Herzinsuffizienz Würzburg, Am Schwarzenberg 15, 97078 Würzburg, Germany

**Keywords:** Epidemiology, Germany, Heart failure, Incidence, Prevalence

## Abstract

**Background:**

Chronic heart failure (HF) is associated with significant healthcare expenditure, morbidity, and mortality. This study investigated the epidemiology of HF in Germany.

**Methods:**

This retrospective study used anonymous healthcare claims data from the German Health Risk Institute on individuals with statutory health insurance. Patients with uninterrupted data from 1 January 2009 to 31 December 2013 or death (whichever occurred first), and ≥2 recorded HF-related diagnoses in 2011, were included. Patients with newly diagnosed HF were identified. Patients were followed up for 2 years from first diagnosis.

**Results:**

Of 3,132,337 eligible patients, 123,925 (55.0% women; mean age 76.2 years) had HF: a prevalence of 3.96%. Of these, 26,368 had newly diagnosed HF: an incidence of 655/100,000 persons at risk. Incidence increased with age and was similar regardless of sex. During follow-up, there were 48,159 hospital admissions among newly diagnosed patients (1.8 hospitalizations/patient/2 years); HF accounted for 6% of these. Additionally, 20,148 patients (16.3%) overall and 5983 newly diagnosed patients (22.7%) died. Most new cases of HF were diagnosed by office-based physicians (63.2%); new cases among hospital inpatients were predominantly diagnosed by internal medicine specialists (70.7%). Overall, 94.0% received their initial prescription for HF treatment from a family practitioner.

**Conclusions:**

The high prevalence and incidence observed in this representative sample emphasize the burden of HF in Germany. Substantial hospitalization rates and mortality highlight the need for early diagnosis and appropriate treatment, and for close cooperation between physician specialties and healthcare sectors.

**Electronic supplementary material:**

The online version of this article (doi:10.1007/s00392-017-1137-7) contains supplementary material, which is available to authorized users.

## Introduction

Heart failure (HF) is a major clinical and public health problem worldwide, associated with significant healthcare expenditure, morbidity, and mortality, particularly in individuals aged ≥65 years [1]. HF was identified as an emerging epidemic as early as 1997 [2]. The prevalence of heart disease increases with age [3]; therefore, HF is set to be a growing public health issue as the population ages.

It has been estimated that up to ~3% of the general population, and 10% of the elderly, may be living with HF in the twenty-first century in the USA [3]. The overall incidence of HF has been estimated to be ~1–2 cases per 1000 individuals, based on data from large, prospective, population-based studies conducted in the USA in the 1990s [4, 5]; however, few studies provide recent estimates of the prevalence and incidence of HF in European populations. A cross-sectional study of individual patient data from Sweden estimated HF prevalence and incidence for the entire Swedish population, adjusting for demographic composition in 2010. The overall prevalence of HF was 2.2%, while the incidence was 3.8/1000 persons at risk [6]. In a cross-sectional, population-based study in Germany, the overall age-standardized prevalence of symptomatic chronic HF was 7.7% for men and 9.0% for women [7]. Finally, in a recent, large, real-world study of data from three German statutory health insurance (SHI) providers, the age- and sex-standardized prevalence of HF was estimated at 1.7% in 2004, 1.9% in 2005, and 1.7% in 2006 [8].

HF is the most common cause of hospital admission and the third most common cause of death in Germany [9]; between 2000 and 2013, the number of hospitalizations for HF increased from 239,694 to 396,380 cases [10]. HF is also associated with a substantial financial burden. The condition accounts for ~1–2% of direct health costs in Western industrialized nations, and ~1.1% of direct health costs in Germany, where the direct hospital costs (€2.9 billion in 2006) are primarily driven by the cost of inpatient stays [9]. A recent systematic review found that direct medical costs for patients with chronic HF were €3417–5576 per patient per year. Furthermore, treatment costs were shown to increase with disease progression [11]. This confirms the high financial impact that HF has on the German healthcare system.

We conducted a retrospective analysis using data from the Health Risk Institute (HRI) database to evaluate the epidemiology of HF in a representative sample in Germany. We further aimed to evaluate selected predictors of the risk of hospitalization and death in patients with HF, and to describe the way in which patients newly diagnosed with HF were diagnosed and treated.

## Methods

### Study design and objectives

This was a retrospective healthcare claims study conducted using data obtained from the German Health Risk Institute (HRI) research database, which contains anonymized data from ~7 million individuals with SHI collected between 2008 and 2013 [12]. Data within the database are provided mainly by company and guild health insurers. Of the 81.2 million inhabitants in Germany, 70.6 million have SHI, while the remaining individuals have private medical insurance [13]; based on this, it has been estimated that the database contains data on ~10% of the population with SHI in Germany.

The HRI database is updated on a monthly basis and provides a complete data set for each patient across all available health care sectors (ambulatory and hospital care; pharmaceuticals; medical aids and remedies; sick leave). Information on the utilization of services on an individual basis enables patient-level analysis of disease and treatment data. For this study, a subset of ~4 million individuals from the 7 million in the database was selected based on the age and sex distribution of the German population as of 31 December 2011 [12].

The specific objectives of this study were to: characterize the study population by age, sex, and New York Heart Association (NYHA) functional classification; estimate the prevalence and incidence of HF by NYHA classification; determine the most common comorbidities; assess the rates of and most common reasons for hospitalization; determine the influence of age on the risk of hospitalization; assess mortality in the 2 years after an initial diagnosis of HF; and identify the physician specialities responsible for HF diagnosis and prescription of HF treatment (including glycosides, angiotensin II receptor blockers, angiotensin-converting enzyme inhibitors, diuretics, β-blockers, calcium-channel blockers, other antihypertensive medication, and other heart medication).

All patient data in the HRI database were anonymized and were collected from individuals receiving routine treatment; therefore, patient consent and approval by an independent local ethics committee were not required.

### Study population

To be eligible for inclusion in this analysis, patients were required to have uninterrupted HRI data from 1 January 2009 to 31 December 2013, or until death (whichever occurred first). Individuals who died before 1 January 2011 were excluded. Patients were required to have at least two documented HF-related diagnoses, according to the International Classification of Diseases and Related Health Problems, Tenth Edition, German Modification (ICD-10-GM), made in either a hospital or an ambulatory setting during the identification period (1 January−31 December 2011). This population is referred to as the total population with HF (Table 1 shows the ICD-10-GM codes that were used to determine which patients were included in this study population). The quarter of the calendar year in which the first HF diagnosis occurred was defined as the index quarter. A subgroup of the total population in whom the onset of HF was considered de novo was identified, based on the absence of a documented HF-related diagnosis in the 1 year preceding the index quarter. These patients are described as those with newly diagnosed HF.

**Table 1:** ICD-10-GM codes used to include patients in the total population with heart failure

ICD-10-GM code	Corresponding diagnosis
I50.0	Right ventricular failure
I50.00	Primary right ventricular failure
I50.01	Secondary right ventricular failure
I50.1	Left ventricular failure
I50.11	NYHA class I
I50.12	NYHA class II
I50.13	NYHA class III
I50.14	NYHA class IV
I50.19	NYHA class not specified
I50.9	Heart failure, unspecified
I11.0	Hypertensive heart disease with (congestive) heart failure
I13.0	Hypertensive heart and renal disease with (congestive) heart failure
I13.2	Hypertensive heart and renal disease with both (congestive) heart failure and renal failure

*ICD*-*10*-*GM* International Classification of Diseases and Related Health Problems, Tenth Edition, German Modification, *NYHA* New York Heart Association

All patients were followed for 2 years after the index quarter. Specific NYHA classes were attributed to patients according to ICD-10-GM coding available in the HRI database. If a patient had more than one NYHA class documented, the last NYHA class documented in 2011 was used. Patients without ICD-10-GM indicators for NYHA class were assigned to a group named ‘any other’.

### Study outcomes

Data collected in the HRI database included: patient demographics comprising age, sex, date of death, region or place of residence, and dates of insurance start and end; indices of outpatient care including ICD-10-GM codes; hospital admission and discharge dates; and diagnoses/reasons for hospitalization. The healthcare sector in which patients received a new diagnosis of HF was examined. The sector in which newly diagnosed patients surviving each quarter were treated was also analysed. The database does not include data on symptom scores, quality of life, or clinical parameters, such as left ventricular ejection fraction or laboratory test results.

Comorbidities were based on main and secondary hospital discharge diagnoses, as well as ambulatory diagnoses; if a patient had one or more diagnoses in the index quarter, these were recorded as comorbidities. Comorbidities were quantified using the Charlson comorbidity index [14].

### Data analysis

Incidences and prevalences are reported as (a) absolute frequencies and (b) frequencies per 100,000 individuals covered by German SHI. Estimates of frequencies in the German SHI population are based on adjustment weighting (simple cell weighting) for the multivariate distribution of age class, sex, and region (federal state level) in Germany.

A logistic regression model with age as a predictor variable was used to estimate the effect that age had on the risks of hospitalization and death. The analyses were adjusted for treatment, dose used, duration of treatment, sex, comorbidities, and Charlson comorbidity index [14].

The absolute number of cases of HF in the HRI sample database was validated against benchmark data from the German Federal (Social) Insurance Authority [Bundesversicherungsamt (BVA)] by adjusting the list of ICD-10-GM codes defining HF in accordance with the BVA definition for the year 2011 [15]. The proportion of ambulatory or hospital outpatient I50-related diagnoses in the database (4.03%) was consistent with BVA data (4.36%) [16]. The frequency of I50-related hospitalizations in the database (2.05%) was compared with numbers reported by the German Federal Statistical Office (2.02%) [17]. Numbers of prescriptions of pharmacological treatments for HF were compared with figures reported in the German drug prescription report [18]. These validations demonstrated that extrapolation of data from the database to general patients with SHI was robust. Statistical analysis was conducted in accordance with the German Guidelines and Recommendations to Assure Good Epidemiological Practice and the guideline Good Practice Secondary Data Analysis [19, 20].

## Results

Of the 4,088,854 patients in the representative subset of the database used, 3,132,337 had observable data from 1 January 2009 to 31 December 2013, had not died before 1 January 2011, and were, therefore, included in the study.

### Prevalence

**Fig. 1:**
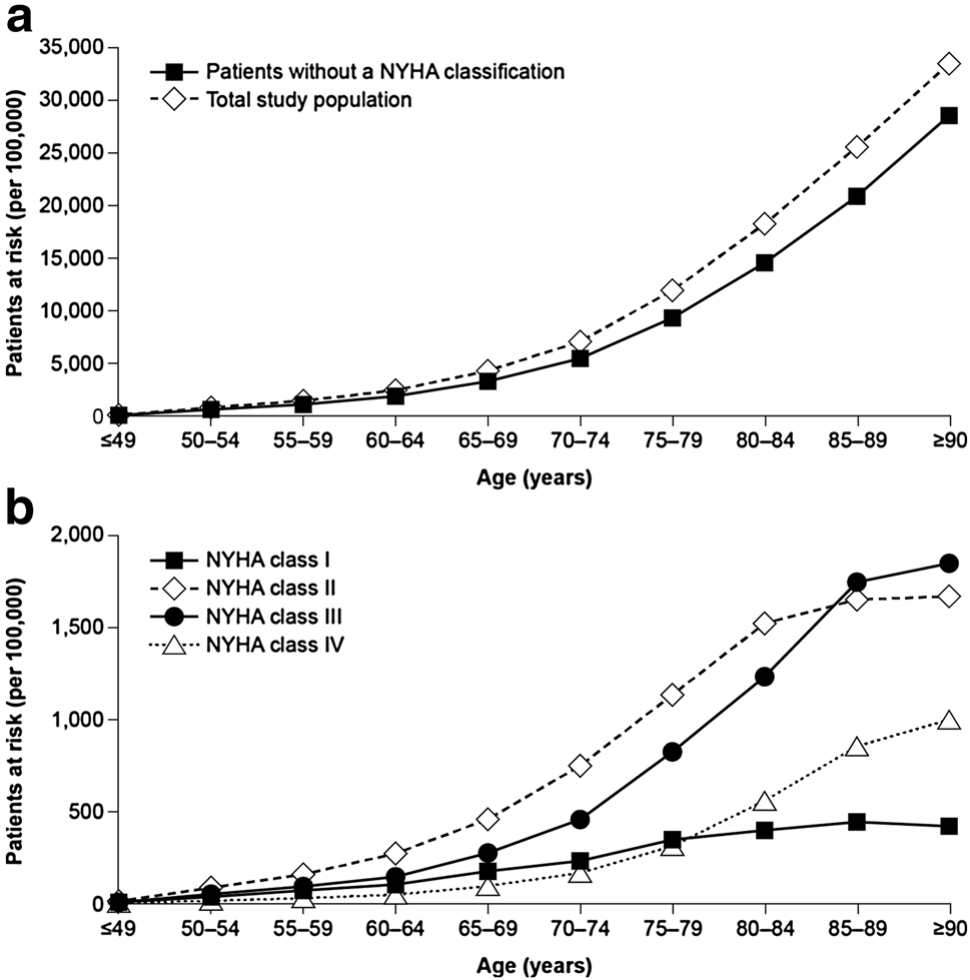
Prevalence of heart failure diagnoses by age group for **a** the total population with heart failure, and **b** the population according to specified NYHA classification. *NYHA* New York Heart Association

In total, 4.0% of patients (*n* = 123,925) included in this study had at least two ICD-10-GM codes indicative of HF recorded in 2011. After adjustment weighting based on age class, sex, and region, this corresponded to an estimated prevalence of HF of 3.9% in the German SHI population. Overall, the mean age of the total population with HF was 76.2 years, and 55.0% were women. In general, the prevalence of HF increased with age, as did the prevalence of HF defined by a specific NYHA class (Fig. 1a, b), reported for 20.9% of patients (25,863 of 123,925 cases).

### Incidence

In total, 26,368 patients in the sample were newly diagnosed with HF in 2011. This corresponds to an overall incidence of 655 new cases per 100,000 persons at risk, and, when extrapolated to the German SHI population, to 524,000 new HF cases annually. The overall incidence was similar in women and men (665 per 100,000 persons at risk vs 645 per 100,000 persons at risk), and for both sexes, the incidence of new cases increased with higher age (Table 2). When categorized by age, the incidence per 100,000 persons at risk was higher in men than in women across all age groups (Table 2).

**Table 2:** Patients with newly diagnosed heart failure in 2011 by age, sex, and NYHA class

Sex	Age, years	NYHA I		NYHA II		NYHA III		NYHA IV		Any other^a^		Total	
		*n*	Per 100,000 persons at risk	*n*	Per 100,000 persons at risk	*n*	Per 100,000 persons at risk	*n*	Per 100,000 persons at risk	*n*	Per 100,000 persons at risk	*n*	Per 100,000 persons at risk
Women	≤49	18	2	37	4	23	2	12	1	234	23	324	31
	50–54	17	10	39	23	17	10	12	7	213	128	298	179
	55–59	26	17	68	45	33	22	14	9	325	214	466	306
	60–64	36	25	139	97	63	44	23	16	544	378	805	559
	65–69	70	61	171	148	91	79	54	47	682	590	1,068	924
	70–74	104	72	353	244	237	164	102	71	1298	899	2094	1450
	75–79	120	113	366	345	297	280	156	147	1543	1456	2482	2342
	80–84	109	141	354	457	332	428	212	274	1665	2148	2672	3448
	85–89	85	170	228	456	290	580	202	404	1321	2642	2126	4251
	≥90	25	106	95	404	119	506	106	450	738	3135	1083	4601
	All	610	30	1850	92	1502	74	893	44	8563	425	13,418	665
Men	≤49	50	5	87	8	33	3	22	2	406	37	598	55
	50–54	45	26	83	47	55	31	20	11	369	210	572	325
	55–59	69	44	148	93	73	46	43	27	502	317	835	527
	60–64	80	52	197	127	104	67	61	39	751	485	1193	771
	65–69	95	80	244	206	147	124	74	62	832	701	1392	1173
	70–74	159	115	416	300	269	194	156	113	1468	1060	2468	1781
	75–79	119	130	414	452	317	346	180	197	1445	1579	2475	2705
	80–84	78	145	269	500	263	489	181	337	1153	2144	1944	3616
	85–89	32	142	147	651	162	717	104	460	652	2886	1097	4855
	≥90	9	124	39	537	39	537	38	523	251	3456	376	5177
	All	736	37	2044	102	1462	73	879	44	7829	390	12,950	645
Total	≤49	68	3	124	6	56	3	34	2	640	30	922	43
	50–54	62	18	122	36	72	21	32	9	582	170	870	254
	55–59	95	31	216	70	106	34	57	18	827	266	1301	419
	60–64	116	39	336	112	167	56	84	28	1295	434	1998	669
	65–69	165	70	415	177	238	102	128	55	1514	646	2460	1050
	70–74	263	93	769	272	506	179	258	91	2766	978	4562	1612
	75–79	239	121	780	395	614	311	336	170	2988	1513	4957	2510
	80–84	187	142	623	475	595	453	393	299	2818	2147	4616	3516
	85–89	117	161	375	517	452	623	306	421	1973	2718	3223	4439
	≥90	34	110	134	435	158	513	144	468	989	3211	1459	4737
	All	1346	33	3894	97	2964	74	1772	44	16,392	407	26,368	655

*n* number of patients with a diagnosis of heart failure in 2011 and no heart failure diagnosis in the previous year; incidence values are based on the number of patients who had continuous data between 1 January 2009 and 31 December 2013, or until death (whichever occurred first), *NYHA* New York Heart Association

^a^ Refers to any patient without a specific NYHA classification

Of the 26,368 patients with newly diagnosed HF, 9976 (37.8%) had received an ICD-10-GM code with the NYHA class specified. The incidence of HF categorized as NYHA class I (33 per 100,000 persons at risk) was lower than the incidence of HF categorized as NYHA class II (97 cases per 100,000 persons at risk), NYHA class III (74 per 100,000 persons at risk), or NYHA class IV (44 per 100,000 persons at risk).

### Hospitalizations and morbidity

**Fig. 2:**
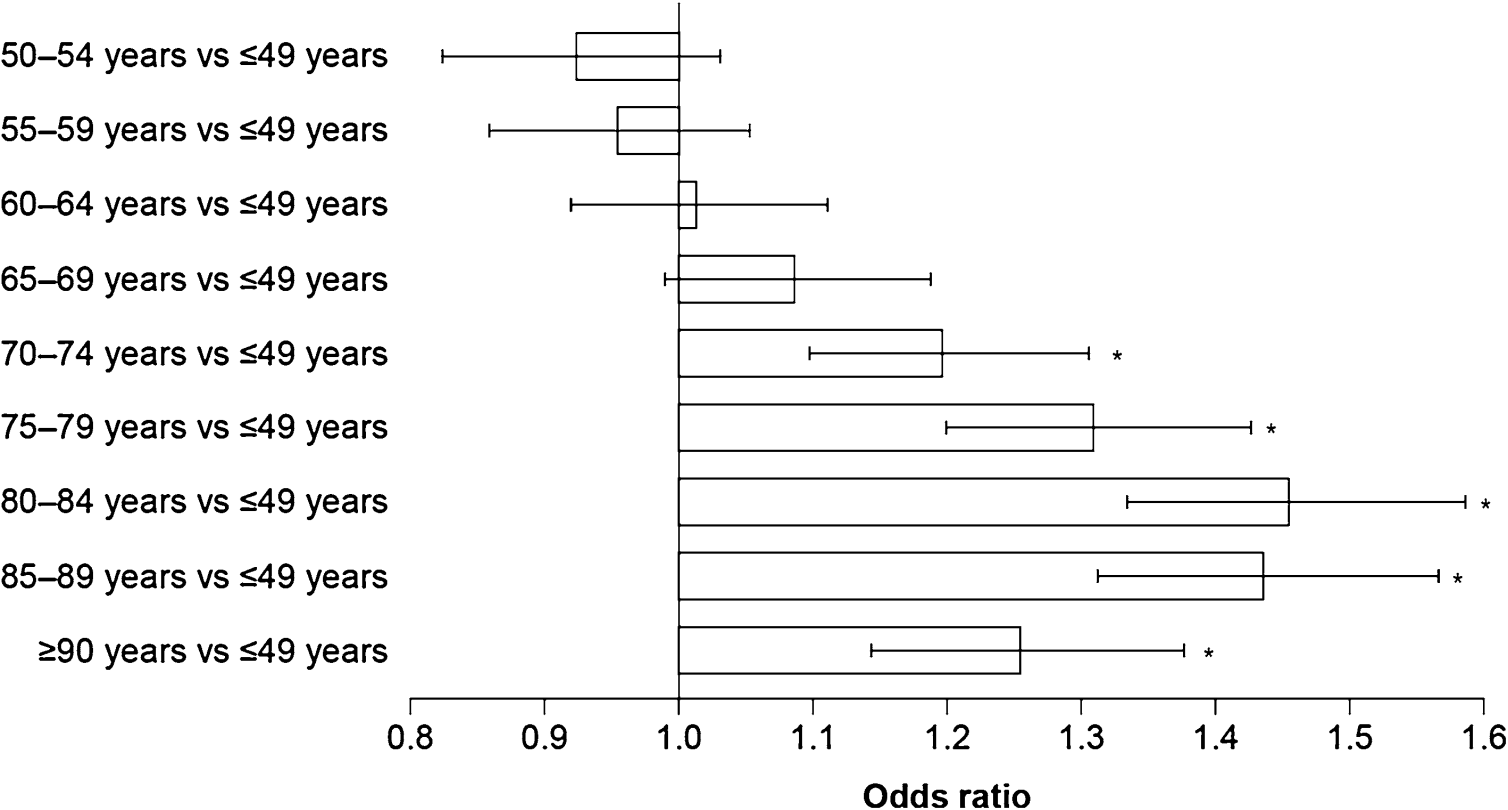
Odds ratios showing the effect of age on the risk of hospitalization in patients with heart failure, estimated using a logistic regression model adjusted for treatment, dose used, duration of treatment, sex, comorbidities, and Charlson comorbidity index. *Error bars* show 95% confidence intervals. *p < 0.0001

In the total population with HF, 68,296 patients (55.1%) were hospitalized over the 2-year follow-up period, and 11,761 of these patients (17.2%) were rehospitalized in the same time frame. Logistic regression modelling showed that there was an increase in the risk of hospitalization with advancing age, with the risk being significantly greater in patients aged ≥70 years than in patients aged ≤49 years (Fig. 2).

Of the 26,368 patients with newly diagnosed HF, the total number of hospital admissions registered in the 2 years after the first HF diagnosis was 48,159, corresponding to a mean of 1.8 admissions per patient. More than two-thirds (73.0%) of these hospital admissions were not related to cardiovascular events; 21.0% were for treatment of a cardiovascular disease other than HF; hospitalization due to HF accounted for 6.0% of total admission counts. Of the patients with newly diagnosed HF, the most frequently recorded specific reason for hospitalization was HF (*n* = 1970 of 26,368 patients; 7.5%; Table 3). The most frequently recorded comorbidities in the index quarters in patients with newly diagnosed HF were primary hypertension (81.7%), dyslipidaemias (50.4%), and chronic ischaemic heart disease (44.4%; Table 4).

**Table 3:** The ten most common reasons for hospitalization in the 2 years following the first diagnosis of heart failure in 2011 in the population with newly diagnosed heart failure (*N* = 26,368)

ICD-10-GM code	Diagnosis	*n*	%
I50	Heart failure	1970	7.47
R06	Breathing abnormalities	1682	6.38
I20	Angina pectoris	1009	3.83
I25	Chronic ischaemic heart disease	917	3.48
I48	Atrial fibrillation and flutter	916	3.47
R10	Abdominal and pelvic pain	766	2.91
R55	Syncope and collapse	733	2.78
J18	Pneumonia, organism unspecified	625	2.37
I70	Atherosclerosis	605	2.29
I63	Cerebral infarction	596	2.26

*n* number of hospitalization events, *ICD*-*10*-*GM* International Classification of Diseases and Related Health Problems, Tenth Edition, German Modification

**Table 4:** The 20 most common comorbidities recorded in the index quarter in which patients were first diagnosed with heart failure (*N* = 26,368)

ICD-10-GM code	Comorbidity	*n*	%
I10	Essential (primary) hypertension	21,530	81.7
E78	Disorders of lipoprotein metabolism and other dyslipidaemias	13,301	50.4
I25	Chronic ischaemic heart disease	11,711	44.4
I11	Hypertensive heart disease	10,313	39.1
E11	Non-insulin-dependent diabetes mellitus	9138	34.7
I48	Atrial fibrillation and flutter	7833	29.7
Z92	Personal history of medical treatment	6661	25.3
M54	Dorsalgia	6577	24.9
N18	Chronic kidney disease	5788	22.0
E66	Obesity	5744	21.8
E87	Other disorders of fluid, electrolyte, and acid–base balance	5096	19.3
Z95	Presence of cardiac and vascular implants and grafts	4965	18.8
J44	Other chronic obstructive pulmonary disease	4795	18.2
E79	Disorders of purine and pyrimidine metabolism	4748	18.0
M17	Gonarthrosis	4525	17.2
H52	Disorders of refraction and accommodation	4432	16.8
I49	Other cardiac arrhythmias	4386	16.6
F32	Depressive episode	4156	15.8
E14	Unspecified diabetes mellitus	3974	15.1
N39	Other disorders of the urinary system	3891	14.8

*n* number of patients, *ICD*-*10*-*GM* International Classification of Diseases and Related Health Problems, Tenth Edition, German Modification

### Mortality

**Fig. 3:**
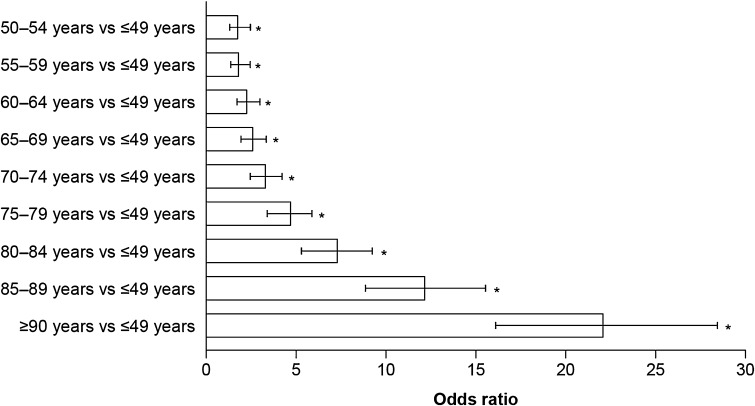
Odds ratios showing the effect of age on the risk of death in patients with heart failure, estimated using a logistic regression model adjusted for treatment, dose used, duration of treatment, sex, comorbidities, and Charlson comorbidity index. *Error bars* show 95% confidence intervals. *p < 0.0001

Among the total population with HF, there were 20,148 deaths (16.3%) in the 2-year follow-up period. There was a significant increase in the risk of death with increasing age, with the risk being significantly greater in patients aged ≥50 years than in patients aged ≤49 years (Fig. 3).

**Fig. 4:**
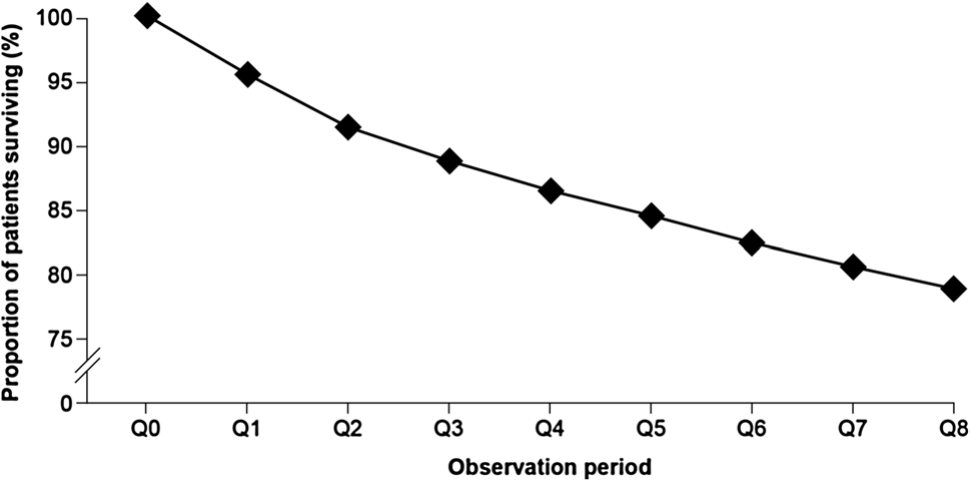
Survival rate among patients with newly diagnosed heart failure in 2011, over the 2-year follow-up period. *Q* quarter

**Fig. 5:**
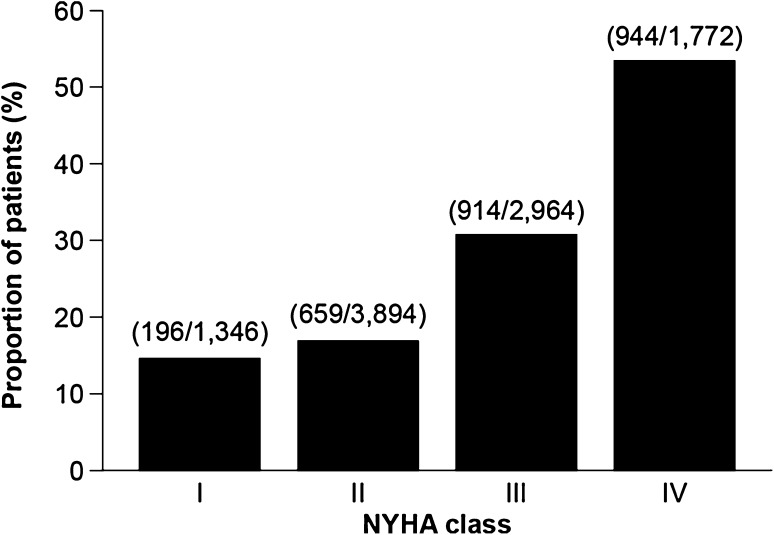
Proportion of patients with newly diagnosed heart failure with a NYHA classification who died within the 2-year follow-up period. The numbers of patients with the NYHA classification who died divided by the total number of patients with that NYHA classification are shown. *NYHA* New York Heart Association

A total of 22.7% of patients (*n* = 5983) with newly diagnosed HF died within the 2-year follow-up period (Fig. 4). Half of the study population who died did so within 9 months of their initial HF diagnosis. Mortality was similar in women and men, and increased with age; a total of 55.9% of individuals aged ≥90 years died in the 2-year follow-up period, compared with 4.0% of patients aged ≤49 years. Mortality also increased with increasing NYHA class: class I, 14.6%; class II, 16.9%; class III, 30.8%; class IV, 53.3% (Fig. 5).

### Diagnosis and prescription

The majority of patients receiving a new diagnosis of HF were identified by an office-based physician (63.2%, *n* = 16,653). In 36.6% (*n* = 9653) of patients, the diagnosis was made in a hospital inpatient setting. Only very few patients were diagnosed in a hospital outpatient setting (0.20%, *n* = 53) (Supplemental Fig. 1).

Most diagnoses made by an office-based physician were made by family practitioners (including general practitioners and general internal specialists and practitioners) (61.6%, *n* = 10,254), but a small proportion (7.0%, *n* = 1171) came from physicians outside family and internal medicine. Furthermore, 6.8% (*n* = 1133) of new diagnoses were made by specialists in internal medicine without a background in cardiology and 14.8% (*n* = 2462) were made by cardiologists. The diagnosis setting of the remaining patients (9.8%, *n* = 1633) was unknown.

The majority (70.7%, *n* = 6827) of new HF diagnoses within a hospital inpatient setting were made by internal medicine specialists; of those, approximately one-quarter (27.3%, *n* = 1864) were made by cardiologists. The remaining diagnoses were made by physicians in surgical (11.7%, *n* = 1131) or other disciplines (13.0%, *n* = 1258), or no information was available (4.5%, *n* = 437). No information was available for new HF diagnoses made in a hospital outpatient setting. Most patients (94.0%) received their initial prescription for the treatment of HF from a family practitioner (Supplemental Fig. 2a), and this proportion remained similar (84.0%) for follow-up prescriptions (Supplemental Fig. 2b). A much smaller proportion of patients received their initial prescription from a physician with a background in cardiology (2%) or other internal medicine (3%); again these proportions remained similar for follow-up prescriptions (cardiology, 5%; other internal medicine, 6%). Data on the professional background of the prescribing physician were unavailable for 4% of patients who received follow-up prescriptions (Supplemental Fig. 2b).

### Validation of the database sample

The estimated prevalence of HF based on the extrapolated number of ambulatory and hospital HF diagnoses, according to the BVA definition adjusted for sex, age, and federal state, was 4.03%; this is consistent with the value of 4.36% reported by the BVA [15]. The estimated proportion of annual hospital admissions due to HF was 2.05% in the HRI database sample; this was similar to the proportion of 2.02% reported by the German Federal Statistical Office (standardized mean difference of 0.25%) [17].

## Discussion

There are few studies providing up-to-date estimates of the prevalence and incidence of HF in Europe [6, 7]; the findings of this large, retrospective database study expand our knowledge in this area and support the notion that estimates of HF in Germany are continuously growing [8]. Considering that over 55% of patients with HF were hospitalized in the 2-year period of the study, and over one-fifth of newly diagnosed patients died within the same time frame, these findings underline the relentless burden of HF in Germany.

This study estimated the prevalence of HF to be 3.96% and the incidence to be 655 new cases per 100,000 persons at risk; this means that 524,000 new cases of HF are expected to be diagnosed per year in Germany. These findings are broadly in line with reports from other European countries and the USA, where prevalence ranges of ~1–3% [3, 7, 8] and incidence ranges of 100–500 per 100,000 persons at risk in the general population, unadjusted for age, have been reported [4, 8].

In our analyses, the prevalence and incidence of HF increased with age, consistent with reports from large population-based studies in the Netherlands [21] and the USA [5]. The incidence of HF was higher in men than in women when examining individual age groups, although this was not the case when looking at overall incidence. This may be explained by the finding that there were more women than men in the older age groups, probably reflecting higher life expectancy in women than in men [22]. The incidence of HF also varied by NYHA classification; the incidence of NYHA class I HF was markedly lower than that of NYHA classes II–IV. This is probably owing to the fact that many patients with NYHA class I HF do not exhibit symptoms attributable to heart disease [23] and therefore remain undiagnosed until symptoms occur.

The financial burden of HF on healthcare systems is dominated by the direct hospital costs of inpatient stays [9, 11], and treatment costs have been shown to increase with disease progression [11]. In the current study, HF was the most common reason for hospitalization of patients with newly diagnosed HF, and the risk of hospitalization increased with increasing age. Importantly, over 80% of these patients suffered from comorbidities that are associated with accelerated disease progression [24]. Furthermore, 16.3% of patients with HF, and over one-fifth of newly diagnosed patients, died within the 2-year follow-up period. As expected, mortality increased substantially with increasing age and NYHA class; more than half of all patients newly diagnosed with HF of NYHA class IV died within 2 years of diagnosis. This underlines the need for appropriate management strategies depending on disease severity; on one hand, palliative strategies for patients with advanced HF need to be carefully considered, but on the other hand, earlier diagnosis and improved management of patients in their most vulnerable phases—immediately after diagnosis and/or discharge from hospital—are needed.

According to our data, office-based family practitioners currently diagnose the majority of patients with HF in the German healthcare system, and remain their principal provider of pharmacotherapy. However, general practitioners face major obstacles in establishing a diagnosis of HF, because they often lack time, expertise, or access to echocardiography [25]. Accordingly, only 20.9% of patients have a NYHA class assigned, which is mandatory to apply guideline-recommended strategies. Because prognosis for patients with HF critically depends on early diagnosis and risk-adapted treatment and care (especially in the 6–12 months after diagnosis), our data strongly underscore the need for more appropriate strategies to apply the evidence-based diagnostic and care algorithms in the German healthcare system. A more systematic involvement of cardiological expertise in diagnosis and in the adoption and up-titration of HF therapy seems intuitive, as does the engagement of dedicated nursing staff (both in hospitals and outpatient practices) to support, accelerate, and amplify such efforts. Further studies, preferably including analyses of regional healthcare providers rather than selected practices, are needed to quantify the effectiveness of patient pathways in terms of costs and patient outcomes.

### Strengths and limitations

Strengths of the current investigation lie in the large sample size and well-validated database it employed. Limitations of this analysis include those inherent to claims database studies, such as the reliance on accurate coding and diagnosis and systematic lack of clinical data. In particular, the proportion of patients with newly diagnosed HF recorded as having NYHA class I disease is probably an underestimation of the true proportion, limiting the conclusions that can be drawn for this subpopulation. A large proportion of initial diagnoses of heart failure were made by general practitioners. However, 55.1% of patients were hospitalized during the 2-year follow-up period, which is likely to involve treatment and/or further tests by a specialist. Furthermore, because ICD-10-GM codes do not differentiate between specific types of HF, it was not possible to determine whether patients had reduced or preserved ejection fraction. Finally, the ~10% of the German population with private medical insurance is not covered in this database, which limits the extrapolation of the results to this population.

## Conclusions

The high prevalence and incidence of HF, the substantial mortality in the 2 years following diagnosis, and the high level of associated morbidity observed in this study underline the heavy burden that HF imposes on the German healthcare system. This highlights the urgent need for improving diagnosis and treatment of this disease. Close collaboration between all physician specialities and healthcare sectors is essential to improve the prognosis for patients with HF in Germany today.

## Electronic supplementary material

Below is the link to the electronic supplementary material.

Supplementary material 1 (PDF 200 kb)
