# Supplementary material for: Epidemiology of heart failure in Germany: a retrospective database study
Source: Clin Res Cardiol. 2017 Jul 26;106(11):913–22. doi: 10.1007/s00392-017-1137-7 (PMC5655572; doi:10.1007/s00392-017-1137-7)
Supplement: Supplementary file 1 — Supplementary material 1 (PDF 200 kb) [file 392_2017_1137_MOESM1_ESM.pdf]

Electronic supplementary material

Epidemiology of heart failure in Germany: a retrospective database study

Clinical Research in Cardiology

Stefan Störk • Renate Handrock • Josephine Jacob • Jochen Walker • Frederico Calado • Raquel Lahoz Stephan Hupfer • Sven Klebs

Corresponding author:
Professor Stefan Störk
Comprehensive Heart Failure Centre Würzburg and Department of Internal Medicine I, University and University Hospital Würzburg, Würzburg, Germany

e-mail address: Stoerk_S@ukw.de

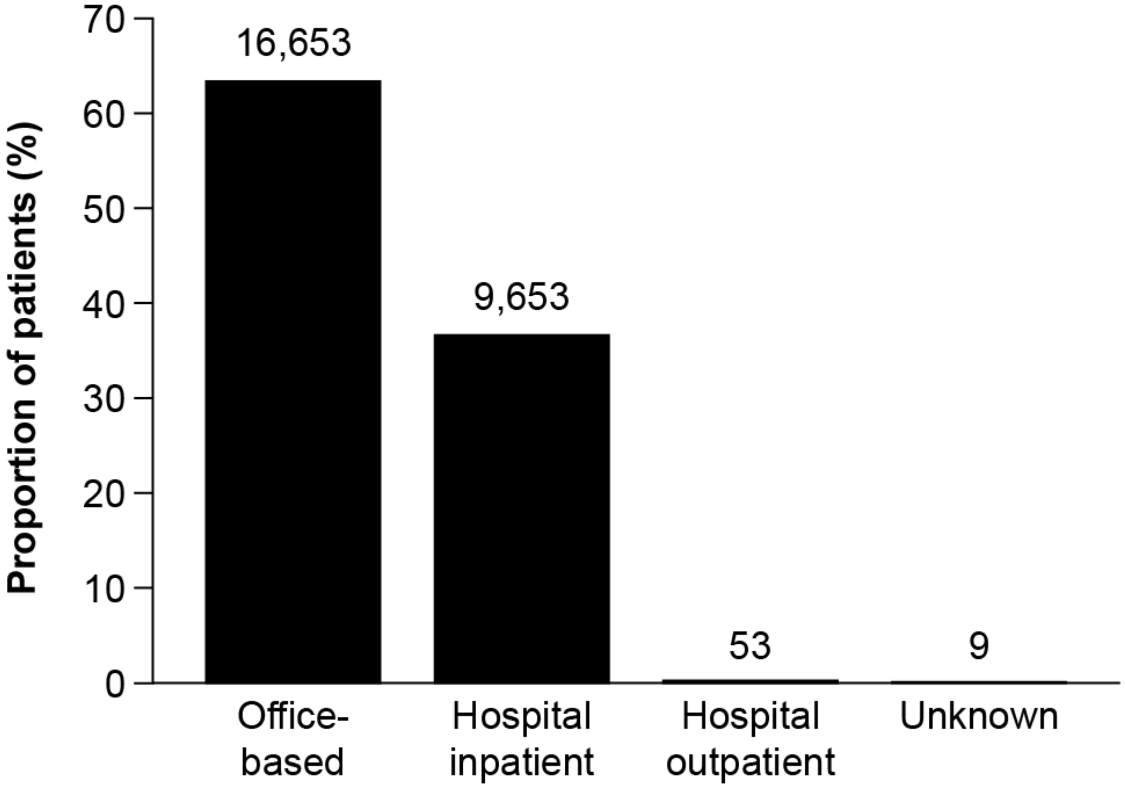

Supplemental Fig. 1 The setting in which patients were newly diagnosed with heart failure

The numbers of patients diagnosed in each setting are shown

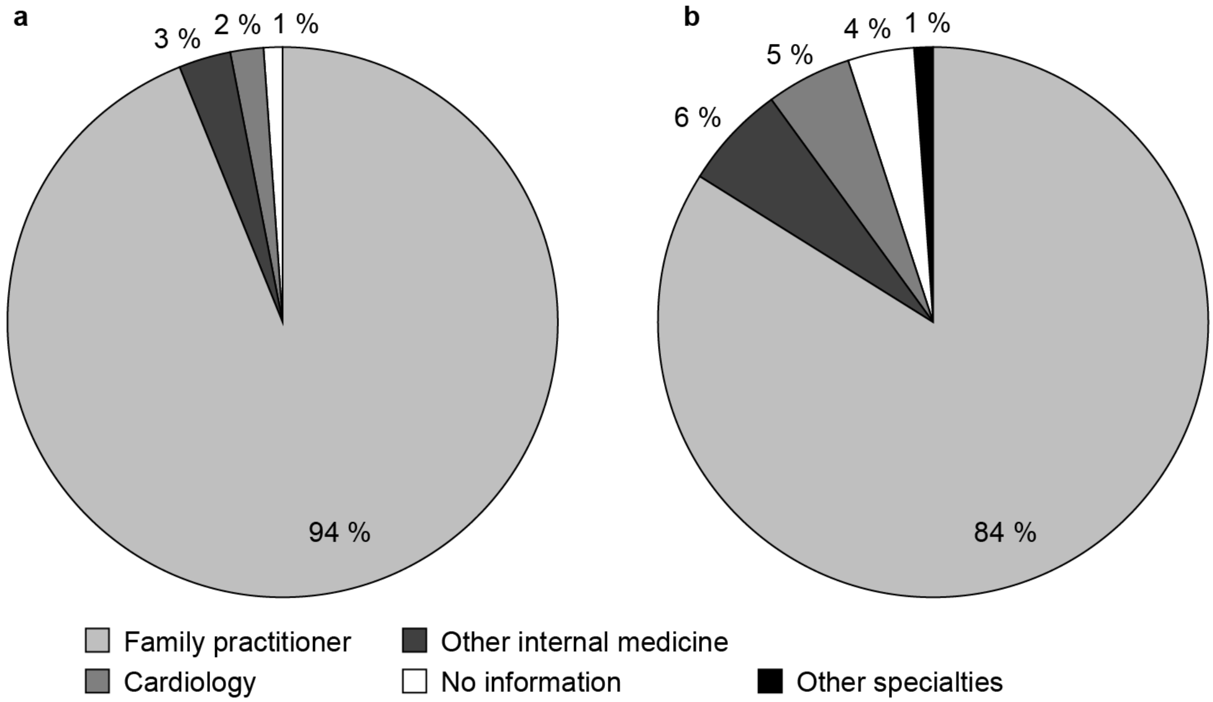

Supplemental Fig. 2 Professional background of physicians providing patients with **a** initial prescriptions and **b** follow-up prescriptions for pharmacotherapy for heart failure
